# Knowledge difference of sexually transmitted infections between Hong Kong undergraduates from local and international secondary schools: A cross-sectional study

**DOI:** 10.3389/fpubh.2022.947932

**Published:** 2022-11-02

**Authors:** Darren Li Liang Wong, Allen Zhang, Kylie K. Y. Cheung, Edmond Pui Hang Choi, May P. S. Lam

**Affiliations:** ^1^Li Ka Shing Faculty of Medicine, The University of Hong Kong, Pokfulam, Hong Kong SAR, China; ^2^School of Nursing, Li Ka Shing Faculty of Medicine, The University of Hong Kong, Pokfulam, Hong Kong SAR, China; ^3^Department of Pharmacology and Pharmacy, Li Ka Shing Faculty of Medicine, The University of Hong Kong, Pokfulam, Hong Kong SAR, China

**Keywords:** sexually transmitted infections, STI knowledge, STI awareness, sex education, secondary schools, undergraduates, university students, youths

## Abstract

**Background:**

Since the delivery of sex education is not standardized across local and international secondary schools in Hong Kong, this study aims to assess and compare the knowledge level of sexually transmitted infections (STIs) between university students who attended local and international secondary schools in Hong Kong.

**Methods:**

From January to March 2019, we conducted a cross-sectional survey among undergraduates at the University of Hong Kong. The primary outcome was STI knowledge as measured by a 29-item quiz. A higher quiz score meant a better STI knowledge level. Students' attitude toward sexual health and their sex education history was collected. Bivariate and multivariate analyses were conducted to evaluate the association factor with a better STI knowledge level.

**Results:**

Three hundred and ninety six students were included in the analysis. Three hundred thirty three (85.35%) students attended local secondary schools and 58 (14.65%) students attended international secondary schools in Hong Kong; 200 (50.51%) students were male and 196 (49.49%) students were female. Compared with students from local secondary school, those from international secondary schools had a significantly higher STI quiz score (18.19 vs. 15.4, *p* = 0.003). The results of multiple linear regression revealed that students in a higher year of study (β = 1.07, *p* < 0.001), from medical faculties (β = 6.96, *p* < 0.001), and from international secondary schools (β = 2.27, *p* = 0.003) achieved a higher STI quiz score.

**Conclusion:**

University students who attended international secondary schools in Hong Kong possess a significantly higher knowledge level of STIs compared with those who attended local secondary schools. Nonetheless, the overall STI awareness among university students is inadequate. The inadequacy of STI awareness calls for the need to plan and implement satisfactory, comprehensive, and standardized sex education across the overall education system in Hong Kong.

## Introduction

Hong Kong is an international city that incorporates both Chinese and Western influences. Values pertinent to traditional Confucianism and Western Christianity continue to shape sexual ideology in Hong Kong (1, 2). Even though Hong Kong is considered the most westernized city in China, sex remains a topic that is rarely discussed openly (3). Nonetheless, attitudes toward sex and patterns of sexual behaviors have been gradually changing in Hong Kong. For instance, the Youth Sexuality Survey of the Family Planning Association of Hong Kong reported that the prevalence of having premarital sex among unmarried males and females aged 18–27 years increased from 35.1 and 27.5% in 1996 to 44.0 and 31.0% in 2006, respectively (4). A similar study conducted in 2011 reported that the majority of unmarried youth (63.8%) held liberal attitudes toward premarital sex, and around half held liberal attitudes toward any forms of sexual activity and premarital pregnancy (4). However, a study found that condom use was suboptimal among university students in Hong Kong, with only 28.46% of students consistently using condoms during sexual intercourse (5). Premarital sex and high-risk sexual behaviors will undoubtedly increase the risk of adverse sexual health outcomes such as sexually transmitted infections (STIs).

STIs remain a major public health issue worldwide (6). Following the changing perceptions and attitudes toward sex, global cases of STIs increased from 486.77 million in 1990 to 769.85 million in 2019 (6). Globally, chlamydia is the most common bacterial STI (7). A study in China reported that the incidence of genital chlamydial infection was 55.32 cases per 100,000 population in 2019; this was higher than the incidence of gonorrhea (9.59 cases per 100,000 population in 2018), suggesting that genital chlamydial infection was a very common STI in China (8). Further, the study reported that the incidence of genital chlamydial infection was higher in females than in males, with a female-to-male ratio of 3.09–1. The highest incidence of genital chlamydial infection was reported in the age group of 20–24 years (184.70 cases per 100,000 population), followed by the age groups of 25–29 years (180.79 cases per 100,000) and 30–34 years (151.53 cases per 100,000 population); these findings imply that young adults are at a higher risk of having genital chlamydial infection (8). In Hong Kong, data on the prevalence and incidence of STIs are limited because STIs are not notifiable locally and no population-based STI prevalence studies are conducted regularly (9). Nevertheless, the first population-based study of chlamydia infections among Hong Kong residents conducted between 2014 and 2016 found that its prevalence was highest in sexually active women aged 18–26 years (5.8%) followed by sexually active men aged 18–26 years (4.8%) (10). The reported prevalence in Hong Kong was higher than the pooled prevalence in the general population (2.9%) as reported by a meta-analysis of 29 studies from 24 countries (11). Regarding the prevalence of STIs in the regions near China, a study in Japan found that the prevalence of asymptomatic chlamydial infection was 8.3% among post-secondary school students (≥ 18 years) (12). Similarly, a study among university students in South Korea reported that the prevalence of chlamydial infection among sexually active men and women was 8.4 and 10.6%, respectively (13). A study among young Thai man reported that the prevalence of chlamydial infection was 7.9% (14). Even though it is difficult to directly compare the prevalence of chlamydia across different studies due to differences in sampling frames, study populations and study time frames, the findings reported from previous studies suggested that chlamydial infection is common. As STIs could be asymptomatic, they would often go unnoticed and may have been further transmitted to other sexual partners (10). Common STIs could lead to severe health complications including pelvic inflammatory disease, ectopic pregnancy, postpartum endometriosis, infertility, and chronic abdominal pain in women (15). STIs could also lead to urethral strictures and epididymitis in men (15). Mother-to-child transmission of STIs can result in complications such as stillbirth, low birth weight, prematurity, and neonatal death (16). Therefore, STIs are a major public health concern that warrant interventions and sex education.

Even though the Hong Kong Education Department issued “Guidelines on Sex Education in Schools” in 1997 to strengthen the implementation of sex education in schools, Hong Kong does not have a mandatory sexual health curriculum (17). Individual schools enjoy flexibility in adopting the approach, contents, and delivery mode of sex education according to their background, mission, ethos, and resources (17, 18). It is similar to the situation in some European countries, such as Italy, where sex education is not mandatory (19, 20). An evaluation of the implementation of sex education in Hong Kong secondary schools revealed a decrease in the percentage of schools having an overall policy on the implementation of sex education owing to a lack of resources and time (17). Moreover, the effectiveness of sex education remains unclear because schools do not assess the learning outcomes (17). Meanwhile, international schools in Hong Kong enjoy greater autonomy in designing and delivering sex education. Furthermore, these schools encourage a holistic education which includes cultural exploration, global perspectives, and whole child development, which could translate to better sexual health awareness and knowledge.

Since the delivery of sex education is not standardized across local and international secondary schools in Hong Kong, we hypothesized that young adults from international schools have a higher knowledge level of STI compared with that of young adults from local schools, resulting in disparities in STI risks among youths. This may warrant the implementation of standardized sex education in all secondary schools across Hong Kong. To the best of our knowledge, no comprehensive and up-to-date study has evaluated the adequacy of STI awareness and quality of sex education of young adults from different secondary school systems. The primary objective of the study is to assess and compare the knowledge level of sexually transmitted infections (STIs) between university students who attended local and international secondary schools in Hong Kong.

## Methods

### Study design and participants

This cross-sectional survey was conducted from January 2019 to March 2019 at the University of Hong Kong (HKU), where undergraduate students who attended secondary schools in Hong Kong were recruited. Convenience sampling was used to recruit study participants. It was not feasible to recruit participants by random sampling in the study setting. Exchange, visiting, and part-time students were excluded from participating in this survey. 396 participants were included in the analysis.

### Ethics

The study protocol was approved by the Institutional Review Board of the University of Hong Kong/Hospital Authority Hong Kong West Cluster (HKU/HA HKW IRB Reference Number: UW 18-650). Informed consent was obtained from each participant.

### Study instrument

The primary outcome was the knowledge level of STIs. A 29-item STI quiz was self-developed with reference to previous studies (21, 22). The total number of correct answers to these 29 questions reflected participants' knowledge level of STIs. Each correct answer was given 1 point, hence the score ranged from 0 to 29, with a higher score indicating a higher knowledge level. The Cronbach's alpha of the knowledge scale was 0.88 in the current sample. The STI quiz is shown in Appendix 1.

We also assessed students' attitude toward sexual health by using the following questions:

- How satisfied were you with the quality of previous formal sex education?
- How would you rate your knowledge of sexual health, especially with regard to STIs?
- How comfortable do you feel in sexual health discussions with peers or friends?
- How comfortable do you feel in sexual health discussions with your parents?

The students were asked to rate on a 10-point numerical scale (from 1 to 10), with a higher score meaning more satisfied/knowledgeable/comfortable.

Further, information on previous sex education, primary source of sex education, and desire for more sex education in secondary schools was collected. Demographic information such as gender, year of study, study faculty, type of Hong Kong secondary school attended, monthly personal expenditure, and religion was collected.

### Data analysis

Descriptive statistics were used to describe the characteristics of the study participants. First, a chi-squared test was used to compare the characteristics between students from local and international secondary schools. Second, an independent sample *t*-test was used to compare the STI quiz score, satisfaction with quality of sex education received, self-rated STI knowledge, comfort level in sexual health discussions with peers and friends, and comfort level in sexual health discussions with parents between students from local and international secondary schools. Third, a multiple linear regression was used to explore the demographic factors associated with the STI quiz score. The model considered factors including gender, year of study, study faculty, type of secondary school, monthly personal expenditure, and religion. Fourth, an independent sample *t*-test was used to compare the STI quiz score between (i) students who received sex education related to topics on STI, contraception, and pregnancy and those who did not; (ii) students whose primary source of sex education was official education and those whose primary source of sex education was the Internet or social media; and (iii) students who desired more sex education and those who did not. Fifth, Pearson's correlation coefficient was used to explore the association between the STI quiz score and satisfaction with the quality of sex education received, self-rated STI knowledge, comfort level in sexual health discussions with peers and friends, and comfort level in sexual health discussions with their parents. Participants with missing data were excluded in a particular analysis. Data analyses were conducted using IBM SPSS Statistics 26.0.

The STROBE checklist was used to report this study.

## Results

Of the 431 students who responded to our self-administered questionnaire, 396 (91.88%) were included in the analysis; 26 (6.03%) who did not study in a Hong Kong secondary school and 9 (2.09%) who attended both a local and an international secondary school in Hong Kong were excluded.

### Descriptive results

Among the 396 students who were included in the analysis, 338 (85.35%) attended local secondary schools and 58 (14.65%) attended international secondary schools in Hong Kong; 200 (50.51%) were male and 196 (49.49%) were female; and 212 (53.54%) studied in medical faculties and 184 (46.46%) studied in non-medical faculties. The students were predominantly in their first four years of studies (*n* = 385, 97.22%). Further, 101 students (25.51%) had a monthly personal expenditure of ≥HK$2,500; 219 students (55.30%) were atheists/agnostic and 104 (26.26%) were religious; 391 students (98.74%) received sex education in secondary schools; 310 (78.28%) received sex education related to topics on STI, contraception, and pregnancy; and 213 (53.79%) desired more sex education in secondary schools. The chi-squared test found statistically significant differences in the monthly personal expenditure and desire for more sex education in secondary school between students from local and international secondary schools. Table 1 shows the characteristics of the study participants.

**Table 1:** Comparisons of characteristics between students from international schools and those from local schools.

	**Total sample**	**Students from local schools**	**Students from international Schools**	***p* ^a^**
	***n =* 396**	***n =* 338**	***n =* 58**	
	***n* (%)**	***n* (%)**	***n* (%)**	
**Gender**				0.105
Male	200 (50.51)	165 (48.82)	35 (60.34)	
Female	196 (49.49)	173 (51.18)	23 (39.66)	
**Year of study**				0.444
Year 1	102 (25.76)	86 (25.44)	16 (27.59)	
Year 2	159 (40.15)	135 (39.94)	24 (41.38)	
Year 3	56 (14.14)	49 (14.50)	7 (12.07)	
Year 4	68 (17.17)	60 (17.75)	8 (13.79)	
Year 5	7 (1.77)	6 (1.78)	1 (1.72)	
Year 6	4 (1.01)	2 (0.59)	2 (3.45)	
**Faculty** ^b^				0.260
Medical	212 (53.54)	177 (52.37)	35 (60.34)	
Non-medical	184 (46.46)	161 (47.63)	23 (39.66)	
**Monthly personal expenditure**				0.005
≤ HK$499	12 (3.03)	10 (2.96)	2 (3.45)	
HK$500–999	60 (15.15)	55 (16.27)	5 (8.62)	
HK$1000–1499	87 (21.97)	79 (23.37)	8 (13.79)	
HK$1500–1999	48 (12.12)	45 (13.31)	3 (5.17)	
HK$2000–2499	80 (20.20)	67 (19.82)	13 (22.41)	
≥HK$2500	101 (25.51)	75 (22.19)	26 (44.83)	
Prefer not to say^c^	8 (2.02)	7 (2.07)	1 (1.72)	
**Religion**				0.161
Religious^d^	104 (26.26)	91 (26.92)	13 (22.41)	
Atheist/agnostic	219 (55.30)	178 (52.66)	41 (70.69)	
Prefer not to say^c^	73 (18.43)	69 (20.41)	4 (6.90)	
**Received sex education in secondary schools**				0.107
Yes	391 (98.74)	335 (99.11)	56 (96.55)	
No	5 (1.26)	3 (0.89)	2 (3.45)	
**Received sex education related to topics on STI, contraception, and pregnancy**				0.837
Yes	310 (78.28)	264 (78.11)	46 (79.31)	
No	86 (21.72)	74 (21.89)	12 (20.69)	
**Desires for more sex education in secondary schools**				0.040
Yes	213 (53.79)	189 (55.92)	24 (41.38)	
No	183 (46.21)	149 (44.08)	34 (58.62)	

This table shows the comparisons of characteristics between students from international schools and those from local schools. The chi-squared test found statistically significant differences in the monthly personal expenditure and desire for more sex education in secondary school between students from local and international secondary schools. There were no statistically significant differences in other characteristics (i.e., gender, year of study, faculty, religion, history of having sex education and history of having sex education related to topics on STI, contraception, and pregnancy).

n, number of participants; %, column percentages (computed by dividing the counts for an individual cell by the total number of counts for the column).

^a^The p-value was were obtained by Chi-squared tests.

^b^Medical faculties include the faculty of medicine and faculty of dentistry. Non-medical faculties include the faculty of science, faculty of arts, faculty of business and economics, faculty of engineering, faculty of education, faculty of social sciences, faculty of architecture and faculty of law.

^c^Students who chose “Prefer not to say” were excluded from the analysis.

^d^Religious people are respondents who selected either “Catholic,” “Protestant,” “Buddhism,” “Islam,” or “Hinduism” for religion in the questionnaire.

### Analytic results

**Figure 1:**
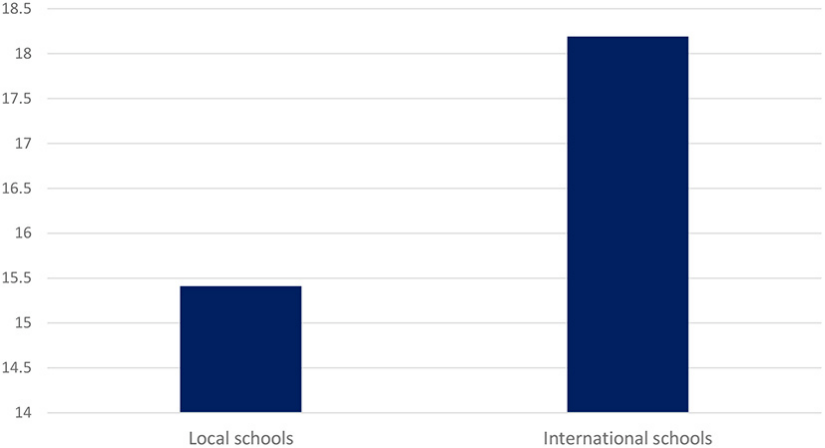
Comparison of sexually transmitted infection quiz score between students from local schools and those from international schools. Compared with students from local secondary schools, those who attended international secondary schools in Hong Kong had a significantly higher STI quiz score (18.19 vs. 15.41, *p* = 0.003). Cohen's d effect size was 0.45.

Compared with students from local secondary schools, those who attended international secondary schools in Hong Kong had a significantly higher STI quiz score (18.19 vs. 15.41, *p* = 0.003). Cohen's d effect size was 0.45. Figure 1 shows the result. In addition, those from international schools rated themselves as more knowledgeable about STIs (6.72 vs. 5.92, *p* = 0.008) and were more comfortable discussing sexual health issues with peers or friends (7.45 vs. 6.24, *p* = 0.008). By contrast, students from local secondary schools were more comfortable discussing sexual health issues with their parents than those from international secondary schools (4.41 vs. 3.55, *p* = 0.008). Table 2 shows the results of the comparison of outcomes between students from local and international secondary schools.

**Table 2:** Comparison of sexually transmitted infection quiz score, satisfaction of sex education received, self-rated STI knowledge, and comfort level in sexual health discussions between students from local schools and those from international schools.

**Knowledge and attitudes**	**Students from local schools**	**Students from international Schools**	***p* ^a^**	**Cohen's d effect size**
	***n* = 338**	***n* = 58**		
	**Mean (*SD*)**	**Mean (*SD*)**		
STI quiz score^b^	15.41 (6.01)	18.19 (6.37)	0.003	0.45
Satisfaction of the quality of sex education received^c^, ^d^	5.84 (1.64)	6.15 (1.99)	0.217	0.17
Self-rated STI knowledge^c^	5.92 (1.80)	6.72 (2.11)	0.008	0.41
Comfort level in sexual health discussions with peers or friends^c^	6.24 (2.11)	7.45 (2.31)	<0.001	0.55
Comfort level in sexual health discussions with parents^c^	4.41 (2.22)	3.55 (2.54)	0.008	0.36

This table shows the results of the comparison of STI quiz score, satisfaction of sex education received, self-rated STI knowledge and comfort level in sexual health discussions between students from local and international secondary schools. Compared with students from local secondary schools, those who attended international secondary schools in Hong Kong had a significantly higher STI quiz score. In addition, those from international schools rated themselves as more knowledgeable about STIs and were more comfortable discussing sexual health issues with peers or friends. By contrast, students from local secondary schools were more comfortable discussing sexual health issues with parents than those from international secondary schools. There were no statistically significant differences in the satisfaction of the quality of sex education received between two groups.

SD, standard deviation; STI, sexually transmitted infections.

^a^The p-value was obtained by independent samples t-tests.

^b^The total number of correct answers to 29 questions reflected participants' knowledge level of STIs. The score ranged from 0 to 29, with a higher score indicating a higher knowledge level.

^c^The students were asked to rate on a 10-point numerical scale (from 1 to 10), with a higher score meaning more satisfied/knowledgeable/comfortable.

^d^n = 380 (Local school, n = 326; International school, n = 54).

The multiple linear regression results revealed that students in a higher year of study (β = 1.07, *p* < 0.001), from medical faculties (β = 6.96, *p* < 0.001), and from international secondary schools (β = 2.27, *p* = 0.003) achieved a higher STI quiz score. Gender, monthly personal expenditure, and religion were not associated with the STI quiz score. Table 3 shows the multiple linear regression results.

**Table 3:** Multiple linear regression to explore the socio-demographic factors associated with the sexually transmitted infection quiz score.

	**STI quiz score^a^**	***p* ^b^**
	**β (95% CI)**	
**Gender**		
Male (reference: female)	0.47 (−0.65, 1.58)	0.414
Year of study^c^	1.07 (0.56, 1.58)	<0.001
**Faculty**		
Medical faculties (reference: non-medical faculties)	6.96 (5.83, 8.09)	<0.001
**Type of secondary school**		
International school (reference: local school)	2.27 (0.78, 3.77)	0.003
**Monthly personal expenditure** ^c^	0.19 (−0.17, 0.55)	0.305
Religion		
Atheists/agnostic (reference: religious)	−0.04 (−1.22, 1.15)	0.948
*R* ^2^	35.37%	

This table shows the multiple linear regression results. Students in a higher year of study, from medical faculties, and from international secondary schools achieved a higher STI quiz score. Gender, monthly personal expenditure, and religion were not associated with the STI quiz score.

β, Unstandardised Coefficients; CI, confidence interval; STI, sexually transmitted infections.

^a^The total number of correct answers to 29 questions reflected participants' knowledge level of STIs. The score ranged from 0 to 29, with a higher score indicating a higher knowledge level.

^b^The p-value was obtained by the multiple linear regression. Three hundred seventeen participants were included in the analysis. Students with missing values were excluded from the analysis.

^c^Continuous variables.

Students who received sex education related to topics on STI, contraception, and pregnancy achieved a higher STI quiz score than those who did not (16.25 vs. 14.24, *p* = 0.024). Cohen's d effect size was 0.30. There were no statistically significant differences in the STI quiz score (i) between students whose primary source of sex education was the Internet or social media and those whose primary source of sex education was official education programmes, and (ii) between student who desired more sex education in secondary schools and those who did not. Additionally, a higher satisfaction with the quality of sex education received (correlation coefficient = 0.12, *p* = 0.016), higher self-rated STI knowledge (correlation coefficient = 0.37, *p* < 0.001), and a higher comfort level in sexual health discussions with peers or friends (correlation coefficient = 0.20, *p* < 0.001) were correlated with a higher STI quiz score (*p* < 0.05). Table 4 shows the results.

**Table 4:** Association between sexually transmitted infection quiz score and parameters of previous sex education and attitudes toward sexual health education.

	**STI quiz score^a^**	***p* ^b^**	**Cohen's d effect size**
	**Mean (SD)**		
**Received sex education related to topics on STI, contraception, and pregnancy**			
Yes (*n =* 310)	16.25 (5.61)	0.024	0.30
No (*n =* 86)	14.24 (7.56)		
**Primary source of sex education**			
Official education^c^ (*n =* 200)	15.83 (6.13)	0.683	0.05
Internet/social media (*n =* 127)	16.11 (5.89)		
**Desire for more sex education**			
Yes (*n =* 213)	15.37 (6.49)	0.114	0.16
No (*n =* 183)	16.34 (5.66)		
	**Pearson's correlation coefficient**	***p***
Satisfaction of quality of sex education received^d^ (*n =* 380)	0.12	0.016
Self-rated STI knowledge^d^ (*n =* 396)	0.37	<0.001
Comfort level in sexual health discussion with peers/friends^d^ (*n =* 396)	0.20	<0.001
Comfort level in sexual health discussion with parents^d^ (*n =* 396)	0.05	0.320

This table shows the results of the association STI quiz score and parameters of previous sex education and attitudes toward sexual health. Students who received sex education related to topics on STI, contraception, and pregnancy achieved a higher STI quiz score than those who did not. There were no statistically significant differences in the STI quiz score (i) between students whose primary source of sex education was the Internet or social media and those whose primary source of sex education was official education programmes, and (ii) between student who desired more sex education in secondary schools and those who did not. Additionally, a higher satisfaction with the quality of sex education received, higher self-rated STI knowledge, and a higher comfort level in sexual health discussions with peers or friends were correlated with a higher STI quiz score.

SD, standard deviation; STI, sexually transmitted infections.

^a^The total number of correct answers to 29 questions reflected participants' knowledge level of STIs. The score ranged from 0 to 29, with a higher score indicating a higher knowledge level.

^b^The p-value was obtained by independent samples t-tests.

^c^Official education refers to sex education conducted by teachers, nurses or other professionals.

^d^The students were asked to rate on a 10-point numerical scale (from 1 to 10), with a higher score meaning more satisfied/knowledgeable/comfortable.

## Discussion

The present study was the first to evaluate the difference in STI knowledge between students from local and international secondary schools in Hong Kong. Full-time HKU undergraduate students who attended international secondary schools in Hong Kong had a significantly higher knowledge level of STIs compared with those who attended local secondary schools, even after adjusting for socio-demographic factors. Further, students in a higher year of study and those from medical faculties achieved a higher STI quiz score. This finding was similar to that reported in a study among students from four Italian universities (19).

Similar to the Italian study (19), the overall STI knowledge among our students, regardless of the type of secondary school attended, was unsatisfactory. The average quiz score of undergraduates from local and international secondary schools in Hong Kong was 15.41 and 18.19 out of 29, respectively. In contrast, a local study conducted in 2013 found that unmarried youths in Hong Kong had adequate sex-related knowledge (4). The previous study in Hong Kong found that on average, young adults correctly answered 10 of 12 knowledge items, with higher levels of knowledge concerning STI transmission and lower levels of knowledge about contraception (4). However, owing to the diverse questions used in different studies to assess the STI knowledge of young adults, it is difficult to objectively compare the knowledge level of STI reported in our study with that of others. Nevertheless, the 12-item sex-related knowledge questions used in the previous study were relatively generic and not comprehensive (4) compared to the questions used in the current study, which encompassed six common STIs: chlamydia, genital herpes, gonorrhea, hepatitis B, HIV, and HPV (21). The low STI knowledge found in the current study strongly indicates the need to improve sex education in secondary schools or even in universities in Hong Kong.

In fact, schools are an ideal setting for delivering comprehensive sex education because school authorities can regulate many aspects of the learning environment to make it more supportive and protective (9). The school setting can also provide an environment in which sex education can be delivered in a developmentally relevant sequence over several grades, thereby building knowledge successively (9). Comprehensive sex education delivered appropriately can clearly empower young people to protect themselves physically, mentally, and emotionally. Offering comprehensive sex education programs in schools can have positive impacts on students, including delays in the initiation and reduction in the frequency of sexual intercourse, a reduction in the number of sexual partners, and an increase in condom use (23). In fact, the “Guidelines for Health Education in Primary and Secondary Schools” released in December 2008 by China's Ministry of Education mandated that the discussion of premarital sex should be introduced into secondary schools. Topics related to sexual victimization were also integrated into sex education to develop adolescents' situational awareness about potential threats and how to identify risk factors. A comprehensive approach to STI prevention education is also included (24). Further, the professional consensus is that good sex and relationship education programmes should start in primary school (25). Therefore, efforts must be taken to overcome obstacles in delivering effective and standardized sex education in the overall education system in Hong Kong to improve the sexual health knowledge and practice of students.

Students from the two different school systems exhibited significant differences in the comfort level of sexual health discussion with peers and friends. Compared with students from local schools, those from international schools felt more comfortable in such discussions. One possible explanation is that international schools have a more liberal and westernized study atmosphere. Therefore, students from international schools were more willing to share and discuss sexual health issues with their friends and peers. In contrast, local secondary schools tend to be more conservative about topics related to sex. Therefore, students from local secondary schools might be embarrassed and reluctant to talk about sex with their peers. We suggest that qualitative studies should be conducted to understand this phenomenon in detail.

In general, students from local and international schools had a low level of comfort (4.41 and 3.55 out of 10, respectively) in discussing sexual health with their parents. In fact, talking about sex remains a taboo in many Chinese families, with most older generations having received no sex education themselves and not knowing how to approach this issue (26). Few Chinese parents are willing to talk about sexual issues with their children (27). A study in China reported that parents were often the sources of sex knowledge for adolescents on less taboo and less sensitive topics such as puberty, but not on more taboo and more sensitive topics such as sexuality and STIs (28). In fact, this phenomenon is not unique to the Chinese culture. Sexuality is also considered taboo in some European countries such as Italy, and discussing it in the family is difficult, especially in southern Italy (19, 20). However, family can have substantial influences on young people's sexual attitudes and expression of sexual behaviors (18). Fundamentally, parents and caregivers can have an important role as their children's primary sexuality educators (23). However, some factors, such as lack of knowledge, skills, and comfort, may impede them in fulfilling this role. Therefore, parents and caregivers must be provided with training and resources to foster discussions of sexual health with their children. For example, the “Talking Parents, Healthy Teens” parenting programme in the United States equips parents and caregivers with the necessary skills to talk about sex with their children (29).

We found that religion was not associated with the STI knowledge level, which is consistent with another study involving university students in Malaysia (22). However, current evidence about the association between religion and STI knowledge level is conflicting. A study among young people in London, United Kingdom, reported that religious students, unlike those reporting no religious affiliation, generally reported lower sexual health knowledge and were more conservative in their attitudes toward sex (30). Socio-cultural values such as religious obstructionism might limit the efficacy of sexual health education, which in turn affects the sexual knowledge and attitude of individuals (19, 20). More studies are needed to further explore the impacts of religion on sexual health outcomes.

In addition to religion and monthly personal expenditure, gender was not a significant predictor of higher STI awareness in this study, which contradicts two American studies which found adolescent girls to have higher STI awareness than boys (31, 32). Similarly, studies among university students in Italy also found that female students had higher sexual-health-related knowledge (19, 20). However, the study instruments used to measure sexual-health-related knowledge varied across different studies, making it difficult to compare our study findings with those of others. More empirical studies should be conducted to explore the gender difference in sexual-health-related knowledge.

We found no significant difference in STI quiz scores between those who primarily learnt about STIs from the Internet/social media and those who learnt from official education. In fact, the Internet and social media are important sources to learn about sexual health. A thematic and critical literature review also reported that adolescents commonly engage with sex information online and are interested in a number of topics, including STIs and pregnancy (33). However, the accuracy and appropriateness of sex knowledge from the Internet and social media are sometimes questionable. Therefore, young people need more guidance to appraise the quality of sex information and knowledge on the Internet and social media. At the same time, the fact that young people popularly obtain sex knowledge from the Internet provides a good opportunity for Internet-based intervention programs. A randomized controlled trial reported that an Internet-based sexual health literacy programme for safe sex practice for female Chinese university students could improve knowledge, attitudes, norms, and self-efficacy regarding condom use (34). Therefore, Internet-based sexual health programmes may be considered to supplement school-based sex education (34).

## Limitations and implications for future studies

This study has several limitations. First, HKU undergraduates are not representative of all youth in Hong Kong. In fact, the disparity and inadequacy of STI awareness among HKU undergraduates revealed in this study further accentuate the disparity and inadequacy of STI awareness among the youth in Hong Kong. Second, further studies should be conducted to understand the impacts of different channels of receiving sexual health education on sexual health knowledge, because young people may receive sexual health education from different channels, not just from schools. This information is important for interventions and health promotion delivery in the future. Third, this study may suffer from recall bias as we recruited undergraduates to provide information about their previous sex education in secondary schools. To minimize recall bias, the multiple linear regression in this study only included objective measures when analyzing the potential factors, including the type of secondary school attended, that could contribute to a higher STI quiz score. Fourth, the STI knowledge quiz contained only 29 question items; therefore, it might not be comprehensive enough to cover all aspects of STI knowledge. We therefore call for the need to develop a psychometrically robust and comprehensive STI knowledge questionnaire. Fifth, we did not collect data on actual sexual behaviors. Sexual knowledge does not necessarily reflect sexual behaviors. Further studies should be conducted to explore the association between sexual knowledge and sexual behaviors. Sixth, the use of convenience sampling might lead to sampling and selection bias. Finally, the causality of different variables cannot be determined owing to the limitation of the cross-sectional study design.

## Conclusion

University students who attended international secondary schools in Hong Kong had a significantly higher knowledge level of STIs compared with that of students who attended local secondary schools. The discrepancy in STI awareness between the two student groups could contribute to disparities in STI risks among the youth in Hong Kong. Nonetheless, the overall STI awareness among HKU undergraduates, as reflected by their STI quiz scores, remains inadequate, which may imply the inadequacy of STI awareness among the general youth in Hong Kong. The inadequacy of STI awareness calls for the need to plan and implement satisfactory, comprehensive, and standardized sex education in the overall education system in Hong Kong. Besides, multi-agency approaches involving different parties such as the government, schools, health care professionals, social workers, families and mass media may be considered to deliver sex education. This would maximize the coverage of sex education among Hong Kong youth and minimize the disparities in STI risks. Through the implementation of effective and standardized sex education, all Hong Kong youth can hopefully be equipped with sufficient sexual health knowledge, including awareness of STIs. This would ultimately reduce the spread of STIs, thereby alleviating the disease burden of STIs. Regarding future research, first, further multicentre studies covering the rest of China for comparison should be conducted to increase the statistical power of studies and to provide more information about sexual health knowledge and attitudes among Chinese populations. Second, qualitative studies should be considered to explore how sexual health is discussed within the family. Third, future studies should explore the relationship among sexual health knowledge, sexual behaviors and STI incidence.

## Data availability statement

The raw data supporting the conclusions of this article will be made available by the authors, without undue reservation.

## Ethics statement

The study protocol was approved by the Institutional Review Board of the University of Hong Kong/Hospital Authority Hong Kong West Cluster (HKU/HA HKW IRB Reference Number: UW 18-650). The participants provided their written informed consent to participate in this study.

## Author contributions

DW, AZ, and KC conceived and designed the study with inputs from ML, coordinated and conducted data collection. DW, AZ, and EC analyzed the data. DW and EC wrote the first draft of the manuscript. All authors interpreted the data and critically revised the manuscript and approved the final version of the manuscript for publication.

## Conflict of interest

The authors declare that the research was conducted in the absence of any commercial or financial relationships that could be construed as a potential conflict of interest.

## Publisher's note

All claims expressed in this article are solely those of the authors and do not necessarily represent those of their affiliated organizations, or those of the publisher, the editors and the reviewers. Any product that may be evaluated in this article, or claim that may be made by its manufacturer, is not guaranteed or endorsed by the publisher.
