# Supplementary material for: Knowledge difference of sexually transmitted infections between Hong Kong undergraduates from local and international secondary schools: A cross-sectional study
Source: Front Public Health. 2022 Nov 2;10:947932. doi: 10.3389/fpubh.2022.947932 (PMC9667946; doi:10.3389/fpubh.2022.947932)
Supplement: Supplementary file 1 [file Table_1.docx]

| 1. | Oral contraceptive pills (口服避孕藥) can protect against STIs. |
| --- | --- |
| 2. | You can get an STI from giving or receiving oral sex. |
| 3. | HIV (Human Immunodeficiency Virus) can be transmitted by sharing of food, drinks or toilets. |
| 4. | You can only get the same STI once and won’t be infected again in the future. |
| 5. | Having multiple sex partners increases the chance of getting STIs. |
| 6. | Female tends to have higher risk of STIs than male. |
| 7. | HIV is not fatal, so treatment is unnecessary. |
| 8. | Genital Herpes (生殖器疱疹) is caused by the same virus as HIV. |
| 9. | A woman can look at her body and tell if she has Gonorrhea (淋病). |
| 10. | Frequent urinary infections can cause Chlamydia (衣原體感染). |
| 11. | Using two condoms at a time provides more protection against STIs than only using one. |
| 12. | It is easier to get HIV if a person has another STI. |
| 13. | HPV is caused by the same virus that causes HIV. |
| 14. | Having anal sex increases a person’s risk of getting Hepatitis B. |
| 15. | Soon after infected with HIV, a person develops open sores on his or her genitals (penis or vagina). |
| 16. | There is a cure for Chlamydia (衣原體感染). |
| 17. | A woman who has Genital Herpes (生殖器疱疹) can pass the infection to her baby during childbirth. |
| 18. | The same virus causes all of the STIs. |
| 19. | HPV can cause Genital Warts (性器疣). |
| 20. | HPV can lead to cancer in women. |
| 21. | A person must have vaginal sex to get infected with Genital Warts (性器疣). |
| 22. | A woman can tell that she has Chlamydia (衣原體感染) if she has a bad smelling odor from her vagina. |
| 23. | There is a vaccine available to prevent a person from getting Gonorrhea (淋病). |
| 24. | If a person had Gonorrhea (淋病) in the past he or she is immune (protected) from getting it again. |
| 25. | HPV can cause HIV. |
| 26. | A man can protect himself from getting Genital Warts (性器疣) by washing his genitals after sex. |
| 27. | There is a vaccine that can protect a person from getting Hepatitis B. |
| 28. | There is a cure for Gonorrhea (淋病). |
| 29. | A man can tell if he has Hepatitis B by feeling his body. |

**Appendix 1: A 29-item STI quiz**
